# Glycosaminoglycans’ Ability to Promote Wound Healing: From Native Living Macromolecules to Artificial Biomaterials

**DOI:** 10.1002/advs.202305918

**Published:** 2023-12-10

**Authors:** Peng Yang, Yifei Lu, Weiming Gou, Yiming Qin, Jianglin Tan, Gaoxing Luo, Qing Zhang

**Affiliations:** ^1^ Institute of Burn Research State Key Laboratory of Trauma Burn and Combined Injury Southwest Hospital Third Military Medical University Chongqing 400038 China; ^2^ Department of Dermatology and Laboratory of Dermatology Clinical Institute of Inflammation and Immunology Frontiers Science Center for Disease‐Related Molecular Network West China Hospital Sichuan University Chengdu 610041 China

**Keywords:** biomaterials, biomimetics, extracellular matrix, glycosaminoglycan, wound healing

## Abstract

Glycosaminoglycans (GAGs) are important for the occurrence of signaling molecules and maintenance of microenvironment within the extracellular matrix (ECM) in living tissues. GAGs and GAG‐based biomaterial approaches have been widely explored to promote in situ tissue regeneration and repair by regulating the wound microenvironment, accelerating re‐epithelialization, and controlling ECM remodeling. However, most approaches remain unacceptable for clinical applications. To improve insights into material design and clinical translational applications, this review highlights the innate roles and bioactive mechanisms of native GAGs during in situ wound healing and presents common GAG‐based biomaterials and the adaptability of application scenarios in facilitating wound healing. Furthermore, challenges before the widespread commercialization of GAG‐based biomaterials are shared, to ensure that future designed and constructed GAG‐based artificial biomaterials are more likely to recapitulate the unique and tissue‐specific profile of native GAG expression in human tissues. This review provides a more explicit and clear selection guide for researchers designing biomimetic materials, which will resemble or exceed their natural counterparts in certain functions, thereby suiting for specific environments or therapeutic goals.

## Graphical abstract

The glycosaminoglycans (GAGs), presented in tissues, participate in all stages of wound healing via binding various functional proteins, assisting signal molecule transmission, supporting cell adhesion, remodeling extracellular matrix (ECM) and other physiological processes. Inspired by the special physicochemical properties and biological activities of GAGs, including GAGs in biomaterials to construct biomimetic materials is a promising approach for improving wound healing.

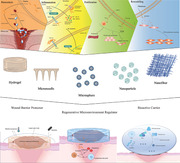

## 1 Introduction

Wound healing, a multifaceted biological process, requires a highly coordinated immune response, cellular proliferation and differentiation, and tissue morphogenesis.^[^ ^1^ ^]^ However, various complex and unfavorable wound microenvironments of the repair process may lead to delayed wound healing and poor healing quality.^[^ ^2^ ^]^ For example, compared with ordinary wounds, burns lead to deeper and thicker skin barrier defects and are more susceptible to microbial infection, resulting in poor wound healing or non‐healing.^[^ ^3^ ^]^ Wounds caused by ulcers from diabetes may experience delayed or no healing due to persistent inflammatory responses, microbial infections, or impaired angiogenesis.^[^ ^4^ ^]^ Thus, the search for medically inclined and clinically beneficial methods to improve the regenerative microenvironment and promote wound healing remains a great challenge.

Currently, there are two biomaterial‐based approaches for controlling skin‐specific regenerative capacity of the body. One approach improves the healing microenvironment by loading and releasing different active molecules to facilitate wound healing.^[^ ^5^ ^]^ The second aids wound healing and functional reconstruction by mobilizing the inherent regenerative potential.^[^ ^6^ ^]^ However, biomaterials that have the ability to activate the intrinsic capacity for regeneration are preferred. Hence, learning the principles of tissue regeneration and repair in nature can help us find the key biomacromolecules involved in the healing process and incorporate them into the construction of biomaterials, benefiting the clinical transformation application of biomaterials. In addition to inheriting adaptable and intricate biological properties, biomacromolecules elevate the likelihood of self‐organization, making multiscale hierarchical behavior predictable.

The extracellular matrix (ECM) plays a crucial role in all processes of wound healing by manipulating the physical and biochemical cues that control cell behavior.^[^ ^7^ ^]^ Glycosaminoglycans (GAGs), the vital components of the ECM,^[^ ^8^ ^]^ are large linear acidic polysaccharides with abundant negatively charged groups, such as carboxyl and sulfonyl groups. GAG processes interact with water or other macromolecules, forming gel‐like networks and binding functional proteins to maintain biomechanical properties of tissues and provide biochemical and mechanical signals that control cell behaviors.^[^ ^9^ ^]^ Participating in the positive mechano‐biological feedback loop between ECM and cells (fibroblasts, macrophages, and stem cells) throughout the tissue regeneration, GAGs mediate biological signals that promote cell growth, migration, differentiation, and apoptosis to control neo‐tissue organization.^[^ ^10^ ^]^ Given their multifaceted roles in preserving the homeostasis of the internal environment, exceptional biocompatibility, inherent biodegradability, and flexible modifiability, GAGs are ideal candidates for advanced biomaterials pertaining to wound healing.^[^ ^11^ ^]^ We searched the PubMed database for articles published in the last five years (2018–2023) using the keywords “glycosaminoglycan,” “hyaluronic acid,” “chondroitin sulfate,” “dermatan sulfate,” “keratin sulfate,” “heparin sulfate,” and “heparin” combined with “wound healing.” Over 1900 articles were retrieved, indicating the extensive exploration of GAGs and GAG‐based biomaterials in wound regeneration and repair. Nevertheless, there are currently few commercial products based on GAGs available for clinic wound repair. The reason for the limited number of products is due to the lack of matching the innate roles and biological activity mechanisms of natural GAGs in in situ wound healing with GAG‐based biomaterials construction and application scenario adaptability, which hinders the understanding of material design and clinical translational application.

To help researchers design GAG‐based biomaterials with more clinical translational potential based on biological and material science principles, we review the innate role of GAGs in different stages of wound healing and reveal the relevant mechanisms by which GAGs perform various functions throughout the healing process. Then, we outline the advantages of different GAG‐based biomaterials, their usefulness, and uniqueness. Moreover, we offer a unique view for deep understanding of the roles of GAG‐based biomaterials in wound healing from three distinct aspects: wound barrier protector, regenerative microenvironment regulator, and bioactive carrier. Finally, we share our insights into the great challenges before the widespread commercialization of GAG‐based biomaterials, including the diverse sources of natural GAGs, principles for the construction of functional dressing, and highlighting the role of GAG‐based biomaterials in regulating and guiding the mechanical environment at different stages of tissue regeneration. Thus, this review provides a more explicit and clear selection guide for researchers designing biomimetic materials, which can resemble or exceed their natural counterparts in certain functions, thereby suiting for specific environments or therapeutic goals.

## 2 Innate Roles of GAGs in Wound Healing

**Figure 1:**
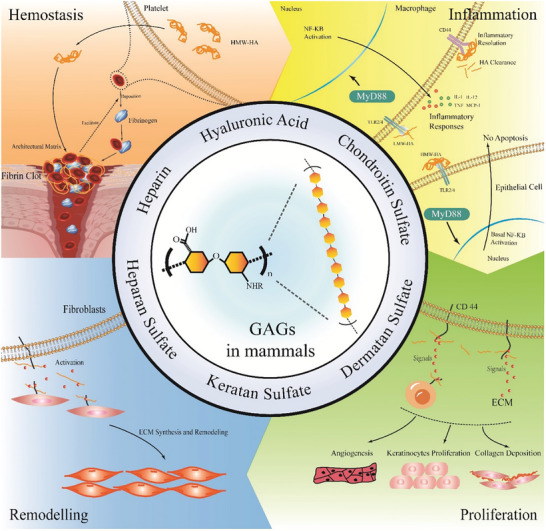
Innate roles and mechanisms of representative GAGs in diverse stages of regeneration. Hemostasis: The HMWHA forms the architectural matrix for deposition of the clotted fibrin to help platelets deposit fibrinogen. Inflammation regulation: LMWHA induces inflammation to stimulate angiogenesis and tissue regeneration, whereas HMWHA regulates the recruitment of inflammatory cells, production of cytokines, and migration of stem cells. Proliferation: GAGs facilitate the interaction between ECM scaffolds and cell receptors by modulating cell–cell and cell–matrix signaling and adhesion, thereby promoting cell adhesion, migration, and proliferation. Remodeling: GAGs facilitate the constant renewal of the ECM to uphold its organizational, structural, and functional integrity by interacting with collagens and glycoproteins. ECM, extracellular matrix; GAG, glycosaminoglycan; HA, hyaluronic acid; HMW, high molecular weight hyaluronic acid; LMW, low molecular weight.

Currently, five main types of GAGs have been identified in mammals, namely, hyaluronic acid (HA), chondroitin sulfate (CS), dermatan sulfate (DS), keratan sulfate (KS), heparan sulfate (HS), and heparin (HEP).^[^ ^12^ ^]^ The ECM of the skin comprises a diverse array of GAGs, with HA and DS being the predominant ones in mature skin. These GAGs substantially contribute to the ability of skin to retain water, thereby preserving its volume and elasticity.^[^ ^13^ ^]^ After skin injury, the body quickly initiates the regeneration and repair processes. Any destruction or prolongation at any stage may hinder wound healing. The GAGs, presented in connective tissues, affect all stages of wound healing through binding with various functional proteins, assisting signal molecule transmission, supporting cell adhesion, and participating in ECM remodeling and other physiological processes.^[^ ^14^ ^]^ **Figure** **1** shows the innate roles and mechanisms of GAGs in posttraumatic hemostasis, inflammation regulation, tissue regeneration, and matrix remodeling.

### 2.1 Hemostasis

Tissue damage‐induced blood vessel rupture and massive bleeding are important risk factors for early mortality of trauma patients. Therefore, rapid and effective hemostasis is a key step that must be addressed immediately after the injury. The spontaneous hemostasis of the body depends on its coagulation function, and GAGs are important regulatory factors involved in this process.^[^ ^15^ ^]^

It is known that HA occurs in multiple forms, chain length being the only distinguishing characteristic between them, and low (LMWHA, *M*_w_ 20–1000 kDa) and high‐molecular‐weight HA (HMWHA, *M*_w_ >1000 kDa) have been shown to have divergent effects on biological activities.^[^ ^16^ ^]^ After a skin injury, clots and platelet aggregates rapidly form, resulting in the transfer of blood to platelet‐rich solid clots that initiate the process of wound healing.^[^ ^17^ ^]^ According to the previous study, fibrinogen is deposited by platelets during the formation of the initial clot. Indeed, fibrinogen is also an HA‐binding protein, and together with the HA‐fibrin complex, helps to maintain a local concentration of HMWHA.^[^ ^18^ ^]^ Meanwhile, the HMW‐HA is considered as the architectural matrix for deposition of the clotted fibrin to facilitate the deposition of fibrin during coagulation. This creates a positive feedback loop to stop bleeding. Thus, levels of HA in its high molecular weight form directly relate to the formation of the initial clot and platelet plug, which are prominent in the earliest stages of wound repair. By contrast, short or LMWHA fragments are highly angiogenic, immunostimulatory, and inflammatory.^[^ ^18^ ^]^ It also reported that controlling molecular weight of HA (1 to 3 × 10^6^ Da) conjugated on biomaterials has been shown a remarkable suppression of platelet activation/aggregation and thrombosis.^[^ ^19^ ^]^ Although the specific mechanism of the above phenomenon was not well understood yet, it is clear that the roles of HA in inducing or suppressing coagulation are primary depend on the molecular weight of HA.

Another important series of natural GAGs, HEP, HS, and DS play a crucial role as negative regulators in the coagulation process. For example, HEP binds to lysine residues of antithrombin III, causing the affinity between antithrombin III and thrombin to increase by 100, leading to immediate thrombin inactivation, and resulting in anticoagulant effects.^[^ ^20^ ^]^ However, the intrinsic glycosaminoglycan neutralization in coagulation control cannot be overlooked, which is an important enlightenment and guiding for the development of GAG‐based biomaterials. A wide range of proteins (platelet factor 4 (PF4), vitronectin, fibronectin, histidine‐rich glycoprotein (HRG), etc.) have been reported to bind and neutralize these GAGs to promote clot formation. Such neutralization can be accomplished via different mechanisms, including neutralizing the negative charge of GAG, interferes with antithrombin binding to immobilized low molecular weight heparin, and promote the internalization and degradation of the GAG‐protein complex, etc.^[^ ^21^ ^]^ Moreover, the artificial modification of GAGs has been widely employed to decrease its anticoagulant activity while preserving its original wound healing promotion properties.^[^ ^22^ ^]^ Therefore, the advanced understanding of special biomolecules and modification strategies that neutralize GAGs (especially HEPs) and subsequently reduce its anticoagulant activity can provide valuable insights for the future development of GAG‐based materials.

### 2.2 Inflammation Regulation

The inflammatory stage typically occurs within minutes of injury, peaks within day 2 to 3, and lasts 4 to 6 d, which creates favorable conditions for regeneration. Chemokines are crucial inducible factors in facilitating the movement of neutrophils from the bloodstream to sites of inflammation. The ECM or GAGs present on the cell membrane facilitate the localization of chemokines to specific sites where inflammatory cells need to be recruited,^[^ ^23^ ^]^ effectively anchoring the soluble chemokine gradient in space and resulting in a prolonged duration of chemotactic signaling. This mechanism aids in the regulation of inflammation.

At the onset of inflammation, GAGs promptly interact with cytokines, chemokines, and growth factors, thereby perpetuating their molecular‐level activity at the injury site and directing the migration of neutrophils and macrophages to the affected areas.^[^ ^11^, ^24^ ^]^ HA, produced by mesenchymal cells, is a critical component of the ECM, reportedly performed as switch of homeostasis and inflammation.^[^ ^25^ ^]^ In quiescent states, HA exists in a large molecular weight form (HMWHA), which support homeostasis and immune surveillance.^[^ ^26^ ^]^ CD44 is the major cell‐surface HA receptor. Under homeostasis, most immune cells show low HA‐binding capacity.^[^ ^27^ ^]^ However, in inflammatory conditions, HA binding with CD44 is specifically upregulated in activated immune cell subsets.^[^ ^28^ ^]^ Meanwhile, the affinity of HA to CD44 depends on its molecular weight and type of target cells.^[^ ^29^ ^]^ It has been proven that the HMWHA inhibits pro‐inflammatory cytokines, and reduced the production of matrix metalloproteins (MMP), proteoglycans, and prostaglandin E2 (PGE2).^[^ ^30^ ^]^ Upon tissue injury and inflammation, HA undergoes degradation mediated by hyaluronidases and nonenzymatically by reactive oxygen species, exhibiting more polydisperse with a lower‐molecular weight form.^[^ ^31^ ^]^ CD44 is responsible for internalization of HA degradation products, which resulting inflammation resolution. However, impaired clearance of HA contributes to persistent inflammation.^[^ ^32^ ^]^ As a sort of damage‐associated molecular patterns (DAMPs), accumulating HA fragments can activate Toll‐like receptor (TLR) 2, TLR4 and their downstream cytoplasmic signal adaptor MYD88 to elicit inflammatory responses of pro‐inflammatory cells.^[^ ^33^ ^]^ Previous studies showed that fragmented HA activated expression and secreting of several cytokines such as plasmogen activator inhibitor‐1 (PAI‐1),^[^ ^34^ ^]^ macrophage inflammatory protein (MIP)−1α, MIP‐1β,^[^ ^35^ ^]^ monocyte chemoattractrant‐1 (MCP‐1), interleukin (IL)−8, and IL‐12 by macrophages.^[^ ^36^ ^]^ And HA with low‐molecular weight (LMWHA) also promotes dendritic cell release cytokines interleukin (IL)−1β, tumor necrosis factor‐alpha (TNF‐α) and IL‐6 via MYD88‐dependent NF‐κB pathway.^[^ ^33^, ^37^ ^]^ In turn, HMWHA protects epithelial cells from apoptosis by maintaining basal level of NF‐κB activation.^[^ ^38^ ^]^

HS/HEP is a GAG known for its anti‐clotting properties. While reperfusion of blood halts the ischemic process by supplying blood and nutrients, it also initiates a chain of events characterized by an inflammatory response, resulting in tissue damage. HS/HEP reportedly reduces or protects against the inflammatory damage caused by ischemia reperfusion.^[^ ^39^ ^]^ Another important role of HS/HEP in the immune system is its ability to interact with proinflammatory cytokines and chemokines. The binding of cytokines to HEP‐like molecules protects them from protease degradation.^[^ ^40^ ^]^ HS/HEP can also bind to and neutralize various mediators released by inflammatory cells, including chemokines, cytokines, and complement factors, to reduce the tissue damage caused by inflammation.^[^ ^41^ ^]^ HEP inhibits the adhesion of neutrophils to endothelial cells, thereby reducing neutrophil overactivation and the release of toxic oxygen radicals and proteolytic enzymes, which contribute to vascular and tissue damage.^[^ ^42^ ^]^

### 2.3 Proliferation

Proliferation occurs days 2 to 10 after injury, in which endogenous fibroblasts and endothelial cells proliferate, and progenitors and stem cells are recruited to the site of injury, leading to the reconstruction of the ECM and angiogenesis.^[^ ^43^ ^]^ During this process, GAGs are related to the interaction between ECM and cells, thus affect cell–cell and cell–matrix communication and cell behaviors.^[^ ^44^ ^]^

GAGs, as the important biological macromolecules in the signaling pathway, promote the binding of growth factors, stabilize their activity, and enhance the interaction of endogenous and exogenous growth factors with their respective receptors.^[^ ^45^ ^]^ The hydrophilicity of HMWHA enables it to maintain a high‐humidity environment, contributes to the formation of porous networks for signaling molecule diffusion and inflammatory cell penetration, and promotes cell proliferation, survival, and nutrient exchange.^[^ ^46^ ^]^ Additionally, HA plays a crucial role in regulating inflammation, cell migration, angiogenesis, and growth factors by interacting with cellular receptors, such as CD44 and intercellular adhesion molecule‐1.^[^ ^47^ ^]^ Therefore, HA is essential for cell adhesion, migration, proliferation, maintenance of cell integrity, and enhancement of intracellular signaling, which are highly beneficial for accelerating tissue repair and regeneration.^[^ ^48^ ^]^ HS interacts with the HEP‐binding domain of fibronectin by linking to the core protein, Syndecan‐4, thereby driving the formation of focal adhesions in concert with integrins,^[^ ^49^ ^]^ providing a stable contact point between cells and the ECM to modulate cell adhesion and migration.^[^ ^50^ ^]^ Additionally, HS assists in the binding of these proteins to growth factors, cytokines, and inflammatory chemokines, thereby regulating cellular migration and proliferation within the ECM.^[^ ^51^ ^]^ HS also activates matrix metalloproteinase (MMP)‐12 through the protein agrin, which guides keratinocytes to the injury site and alter the cytoskeletal architecture after injury. Consequently, this allows the associated cells to counteract unfavorable mechanical stress and accelerate cell migration.^[^ ^52^ ^]^ Small leucine‐rich proteoglycans present in KS can also affect the growth factors that regulate fibroblast adhesion and promote fibroblast contraction.^[^ ^53^ ^]^

Angiogenesis is a crucial process during the proliferation stage and involves the growth of new blood vessels from pre‐existing vessels. Various proangiogenic factors and cell receptors activate endothelial cells, which in turn migrate and proliferate, aiding in the construction of scaffolds and the maintenance of structural integrity.^[^ ^54^ ^]^ The combination of HS/HEP and angiogenic growth factors considerably affects angiogenesis and wound healing.^[^ ^55^ ^]^ The various factors identified to regulate sprouting angiogenesis include VEGF, FGF, transforming growth factor β (TGFβ), and platelet‐derived growth factor B (PDGFB).^[^ ^45^ ^]^ The most well‐known stimulator of angiogenesis is VEGF, regardless of physiological or pathological conditions.^[^ ^56^ ^]^ VEGF signaling activity occurs through binding to the receptor and cellular surface HSPG.^[^ ^57^ ^]^ HSPG modulates impaired angiogenic signaling by controlling VEGFR2 activation.^[^ ^58^ ^]^ CSPG can affect TGFβ and PDGFB signals as well as regulate various steps of angiogenesis. Thus, CS and HS functionally overlap in VEGF‐induced sprouting angiogenesis.^[^ ^59^ ^]^ GAGs create a favorable environment for angiogenesis by directly affecting the expression of angiogenesis‐related cytokines. For instance, the DS/CS chain with decorin controls the formation of type I collagen and interacts with collagen fibrils to form a fibrous network,^[^ ^60^ ^]^ which resists proteolysis to improve the stability of the fibrous network and the elasticity and rigidity of the ECM.^[^ ^61^ ^]^ This provides a favorable environment for angiogenesis.^[^ ^62^ ^]^

### 2.4 Remodeling

Remodeling is the final stage of wound healing, the tissue undergoes enhancement through recombination, degradation, and resynthesis, enabling the healed tissue to fulfill the biophysical requirements of both structure and function. Throughout this process, GAGs play a vital role in maintaining the rigidity and stability of the ECM structure.

During neotissue remodeling, GAGs interact with collagen and glycoprotein to promote the continuous self‐renewal of ECM, thereby recovering its organizational, structural and functional integrity. For example, application of thiol‐functionalized HA derivative strongly inhibited contraction of a collagen matrix, whereas native high molecular weight HA (HMWHA) facilitated collagen gel contraction, suggesting that manipulating the interaction of HA with other matrix molecules can alter ECM remodeling in wound healing.^[^ ^63^ ^]^

HA promotes fibronectin deposition, which builds a temporary ECM, thus initiating and promoting ECM assembly and maturation, and promoting neotissue remodeling.^[^ ^64^ ^]^ On the other hand, the ECM that composed by GAGs provides physical support and mechanical cues for embedded cells, including endothelial cells, macrophages, and myoblasts. For example, in the epidermis, the basal layer is rich in HA, CS and KS, which support the proliferation of basal keratinocytes,^[^ ^65^ ^]^ as well as the migration and proliferation of dermal fibroblasts mediated by HA in the dermis, which are critical for controlling tissue remodeling.^[^ ^66^ ^]^

## 3 Forms of GAG‐Based Biomaterials

**Figure 2:**
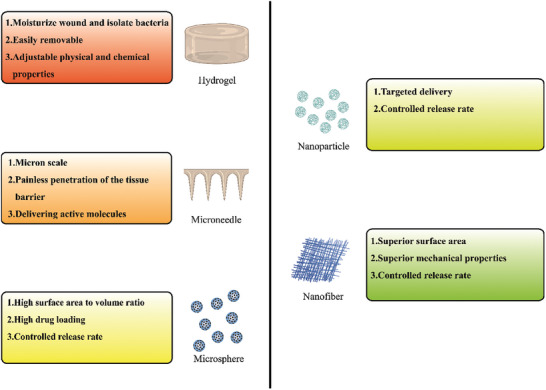
Different forms of GAG‐based biomaterials and main advantages.

Biomaterials provide extracellular biochemical cues and the intracellular delivery of reprogramming factors to direct endogenous cell mobilization and homing dynamics, including diffusion, proliferation, matrix deposition, migration, and tissue regeneration. It is necessary to construct biomaterials derived from GAGs with suitable topological, dynamic viscoelastic and biodegradable properties according to their site of action and corresponding regenerative repair procedures, and to manufacture these biomaterials in various forms, such as nanoparticles, microspheres, scaffolds, hydrogels and microneedles, etc., thus meeting the complex in situ regeneration and repair processes.^[^ ^67^ ^]^ Therefore, selecting appropriate forms and modalities of biomaterials is an essential principle for research on GAG‐based biomaterial in line with the requirements of regenerative medicine.^[^ ^68^ ^]^ The various presentation modalities of biomaterials based on GAGs, and their principal attributes have been outlined in **Table** **1** and **Figure** **2**.

**Table 1:** Diverse manifestations and principal attributes of GAG‐based biomaterials (CS, chondroitin sulfate; GAG, glycosaminoglycan; HA, hyaluronic acid).

Forms	Applicability	Represent GAG‐based materials	Characteristic	References
Hydrogels	Wound dressing; Injectable hydrogel.	Oxidized HA and hydroxypropyl chitosan‐based injectable nanocomposite hydrogels	Moisturizing properties; Easy to remove; Plasticity.	[69]
Microneedles	Colonization; Bioactive carrier.	HA tip, double‐layered microneedles loaded with antimicrobial agents	Micron scale; Painless penetration of barrier.	[70]
Microspheres	Injectable stent; Bioactive carrier.	CS microspheres frameworks	High surface‐volume ratio; High drug loading; Controlled release rate.	[71]
Nanoparticles	Bioactive carrier.	HA nanoparticles	Targeted delivery; Controlled release rate.	[72]
Nanofibers	Directional construction of scaffolds; Bioactive carrier.	Magnesium mineralized antibacterial nanofiber dressing containing CS	Superior surface area; Superior mechanical properties; Controlled release rate.	[73]

### 3.1 Hydrogel

Owing to their beneficial properties, hydrogels are extensively use in wound treatment.^[^ ^74^ ^]^ The microenvironment formed between the wound bed and the hydrogel provides sufficient moisture and facilitates bacterial isolation.^[^ ^75^ ^]^ Additionally, hydrogels can be easily removed from wounds to avoid secondary damage to the healing tissue.^[^ ^76^ ^]^ Hydrogels made from GAG‐based biomaterials are highly versatile for research and applications.^[^ ^77^ ^]^ GAGs possess numerous reactive groups available for modification, which provide a structural basis for the development of GAG‐based hydrogels with superior strength, toughness, and stability.^[^ ^78^ ^]^ Physically cross‐linked hydrogels are formed mainly through physical effects, such as ionic interactions, hydrogen bonds, and chain entanglement.^[^ ^79^ ^]^ Consequently, physically cross‐linked hydrogels are unstable and reversible under heating or other stimuli.^[^ ^80^ ^]^ Nevertheless, it avoids the introduction of toxic chemical cross‐linking agents during the preparation. In contrast, chemically cross‐linked hydrogels are stabilized by covalently cross‐linked networks.^[^ ^81^ ^]^ Initiators, organic solvents, and stabilizers used in chemical cross‐linking may be harmful to the host cells or the system as a whole compromising the safety of wound treatment and potentially leading to infection, difficult healing, or scarring.

**Figure 3:**
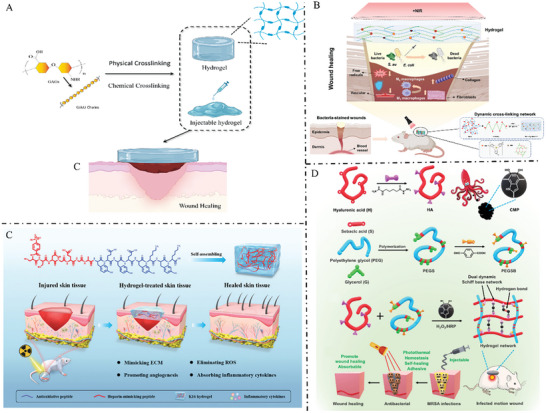
A) GAG‐based hydrogels prepared by physical or chemical cross‐linking promote wound healing. B) Physical dual network cross‐linked photothermal antibacterial multifunctional hydrogel adhesive for wound healing of drug‐resistant bacterial infections. Reproduced with permission.^[^ ^82h^ ^]^ Copyright 2023, Elsevier ltd. C) A heparin‐mimicking peptide hydrogel is applied topically to skin wounds to mimic the ECM, neutralize reactive oxygen species, alleviate inflammation, and stimulate angiogenesis, thereby facilitating the repair of skin injuries.^[^ ^83^ ^]^ Copyright 2023, Wiley‐VCH. D) Preparation of injectable dual dynamic network hydrogel and promoting wound healing of methicillin‐resistant *Staphylococcus aureus* infection. Reproduced with permission.^[^ ^82i^ ^]^ Copyright 2021, Elsevier ltd. ECM, extracellular matrix; HA, hyaluronic acid; ROS, reactive oxygen species.

The hydrophilicity, plasticity, flexibility, biocompatibility, nontoxicity, and biodegradability of GAG enable GAG‐based hydrogels to provide a moist wound environment during tissue regeneration, isolate bacteria to prevent secondary infections, remove exudates from wounds owing to their hydrophilicity, and provide a favorable environment for accelerated angiogenesis and collagen maturation.^[^ ^82^ ^]^ These actions promote tissue regeneration while preventing additional damage. The mechanical properties of GAG‐based hydrogels can be adjusted by tuning the cross‐linking index or pattern to meet the requirements of different wound types and sites. For example, an antioxidant HEP‐mimetic peptide hydrogel self‐assembles into a hydrogel with a 3D mesh‐like porous nanofiber structure, which can provide physical support for skin repair, similar to the ECM. Additionally, the HEP‐mimetic peptide hydrogel can inhibit early wound degradation, reduce inflammatory infiltration, promote angiogenesis, and facilitate collagen deposition, thereby enhancing wound healing.^[^ ^83^ ^]^ GAG‐based hydrogels are malleable in shape, making them suitable for different drug routes. These hydrogels can be accurately injected into deep, enclosed tissue anatomy to repair irregularly shaped lesions.^[^ ^69^, ^82^, ^84^ ^]^ Injectable hydrogels must be biodegradable and biocompatible, which agrees with the properties of GAG‐based biomaterials.^[^ ^85^ ^]^ **Figure** **3** provides a summary of the representative hydrogels based on GAGs.

### 3.2 Microneedles

Microneedles are ultrasmall needles in the micrometer range, whose structure can be highly customized to meet different tissue contact requirements and drug delivery modes.^[^ ^86^ ^]^ Microneedles offer a simple and reliable delivery method for colonization or delivery through tissue barriers during the regeneration and repair of organs or the epidermis, especially in chronic wounds with poor drug permeability due to the presence of biofilms.^[^ ^87^ ^]^

**Figure 4:**
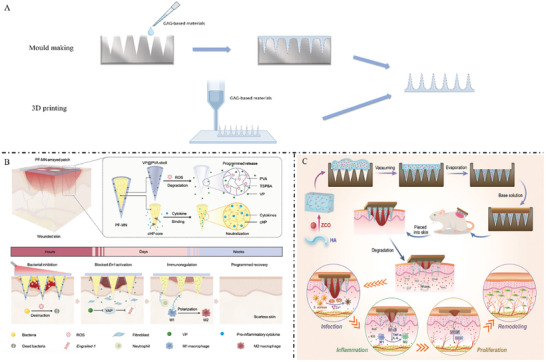
A) Common manufacturing methods of microneedles: mold making and 3D printing. B) A core–shell structured microneedle array patch is designed to combat multidrug‐ resistant bacterial biofilms and promote scar‐free wound repair. Reproduced with permission.^[^ ^87c^ ^]^ Copyright 2023, Springer Nature. C) Schematic diagram of the preparation and application of the multifunctional HA microneedle patch embedded by cerium/zinc‐based composites. Reproduced with permission.^[^ ^89b^ ^]^ Copyright 2023, Wiley‐VCH.

As crucial constituents of the ECM, GAGs possess distinct advantages in the production of microneedles. They are readily recognized and accepted by the body and can be safely metabolized and excreted by the kidneys. Moreover, GAG‐based biomaterials possess a natural affinity for cells and biomolecules, which can aid in the efficient delivery and maintenance of active cargoes in vivo.^[^ ^94^ ^]^ The GAG‐based microneedles are usually used for transdermal delivery of active substances to promote tissue regeneration. Owing to its exceptional biodegradability and bioactivities,^[^ ^88^ ^]^ HA is frequently utilized in the production of soluble microneedles.^[^ ^89^ ^]^ Following drug administration, the soluble microneedle material dissolves, releasing the payload or coating content, and ensuring faster and safer delivery of molecules to a specific location.^[^ ^90^ ^]^ The second category is the use of GAG‐based microneedles as scaffolds for in situ regeneration to enhance the original healing ability of damaged tissues.^[^ ^91^ ^]^ In addition to the aforementioned applications, GAGs themselves can also process direct effects on the subjects as functional factors. For instance, HA microneedle loaded with HEP using a thrombin‐cleavable peptide linker. This patch containing HEP can detect the level of thrombin in blood vessels and autonomously regulate blood coagulation over an extended period.^[^ ^92^ ^]^ Several representative GAG‐based microneedles have been illustrated in **Figure** **4**.

### 3.3 Microspheres

Microspheres typically have a diameter ranging from 1 to 1000 µm, which can be administered via various routes, including oral administration, intravenous or subcutaneous implantation, and intraperitoneal injection.^[^ ^93^ ^]^ The high surface area‐to‐volume ratio of microspheres provides a bridge‐like framework and ample space for cell migration, adhesion and growth.^[^ ^71^ ^]^ Moreover, the special slow‐release function of the microspheres gives the material the characteristics of continuous drug administration, which is facilitating the long‐term administration of severe or chronic wounds.^[^ ^94^ ^]^

**Figure 5:**
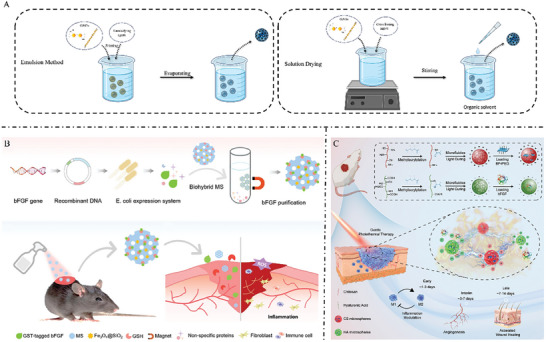
A) Two representative strategies for GAG‐based microspheres preparation. B) Magnetic hybrid microspheres composed of agarose and HA by microfluidic electrospray technology can be widely used in the design of various biomaterials and bioactive compounds. Reproduced with permission.^[^ ^95^ ^]^ Copyright 2021, Elsevier ltd. C) Negatively charged HA microspheres loaded on charge‐driven self‐assembled microspheres hydrogel scaffolds provide a biomaterial for diabetic wound healing. Reproduced with permission.^[^ ^96^ ^]^ Copyright 2023, Wiley‐VCH. MS, microsphere.

The GAG‐based microspheres usually consist of one or more GAGs, and have the ability to sustained release payloads, protect the active factors, and improve the improve bioavailability at the site of action.^[^ ^95^ ^]^ For example, Charge‐driven self‐assembled HA microsphere scaffold has been demonstrated to promote cell proliferation, migration, angiogenesis, and macrophage polarization. Additionally, it accelerates the wound healing process by inhibiting the inflammatory response of the wound and promoting angiogenesis and tissue remodeling.^[^ ^96^ ^]^ HEP‐modified porous microspheres containing PDGFs (Hep‐PMSs) have been shown to reduce the mRNA levels of pro‐inflammatory cytokines and increase the mRNA levels of anti‐inflammatory cytokines, such as IL‐4, IL‐10, and IL‐13 to promote healing.^[^ ^97^ ^]^ Thus, GAG‐based microspheres have potential for applications in clinical practice. **Figure** **5** illustrates the representative preparation and applications of GAG‐based microspheres.

### 3.4 Nanoparticles

Nanoparticles can be transported to remote target sites by binding to biospecific ligands, which can markedly enhance intracellular drug concentration and serve as intracellular drug depots for sustained release.^[^ ^98^ ^]^ An increasing number of multifunctional nanoparticle materials have been developed to provide promising solution for wound healing.

**Figure 6:**
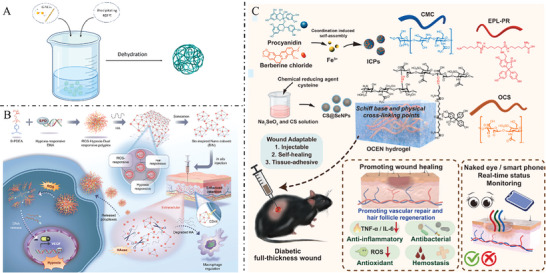
A) Preparation of GAG‐based nanoparticles. B) HA, ROS‐responsive B‐PDEA, and hypoxia‐sensitive VEGF expression plasmids were formulated into nanoparticles by electrostatic interaction, which has a multilevel stimulus response‐ability for ischemic tissue repair and can promote tissue repair after complex injury. Reproduced with permission.^[^ ^102^ ^]^ Copyright 2023, Wiley‐VCH. C) The nanocomposite hydrogel prepared by wrapping chondroitin sulfate modified selenium nanoparticles accelerates wound healing. Reproduced with permission.^[^ ^104^ ^]^ Copyright 2023, Elsevier ltd.

GAG‐based biomaterials are extensively utilized in nanotechnology due to their diverse chemical compositions, charges, and molecular weights.^[^ ^99^ ^]^ The use of GAG helps nanoparticles improves their physical and chemical stability, thus enhancing cell–tissue interactions, controlling drug release, and increasing the bioavailability and efficacy of active substances. Their high hydrophilicity and ability to form noncovalent bonds with biofilms make them highly bioavailable in the blood, which can provide sustained effects and effectively reduce the dose and side effects of the drug.^[^ ^100^ ^]^ Moreover, the nanomaterials incorporating GAGs usually possess corresponding bioactivities. For example, the self‐assembled HA‐NPs have been shown to induce CD44 aggregation, which bears a resemblance to HMWHA containing multiple CD44 binding sites.^[^ ^101^ ^]^ Furthermore, these nanoparticles have exhibited anti‐inflammatory effects via the same mechanism as HMWHA.^[^ ^72^, ^102^ ^]^ A nanosystem with arachnoidal viscosity, fabricated using HA, reactive oxygen species (ROS)‐responsive B‐PDEA, and hypoxia‐sensitive VEGF‐expressing plasmids, has the potential to adhere to damaged or ischemic tissues due to the affinity of HA for the widely expressed receptor CD44. On the other hand, GAGs are versatile carriers that can be loaded with various functional biomolecules to provide nanoparticles with additional functionality. For example, HA‐based nanoparticles containing doxorubicin was employed in targeted therapy to decrease the expression of cell apoptosis and inflammatory markers.^[^ ^103^ ^]^ The nanocomposite hydrogel coated with CS modified selenium nanoparticles can effectively induce skin tissue remodeling, promote the regeneration of blood vessels and hair follicles, remove free radicals from wounds, and accelerate wound healing.^[^ ^104^ ^]^ Some nanoparticles based on GAGs have been illustrated in **Figure** **6**.

### 3.5 Nanofibers

Nanofibers are ultrafine polymer fibers with diameters below 1000 nm obtained through electrospinning techniques. Unlike other common fibrous materials, nanofibers are most characterized by high flexibility, which makes them adequate for handling wounds of different parts and shapes, and can guide the arrangement of cells in the process of wound healing by controlling the shape and arrangement of fibers, and construct anisotropic scaffolds adapted to different wounds. Furthermore, the porous structure of the nanofibers promoted gas exchange and exudate absorption at the wound site, ultimately leading to skin tissue regeneration. Nanofibers can be loaded with therapeutic drugs or active ingredients, and their structure can be adjusted to achieve controlled and slow drug release, which has great potential for skin wound healing.

**Figure 7:**
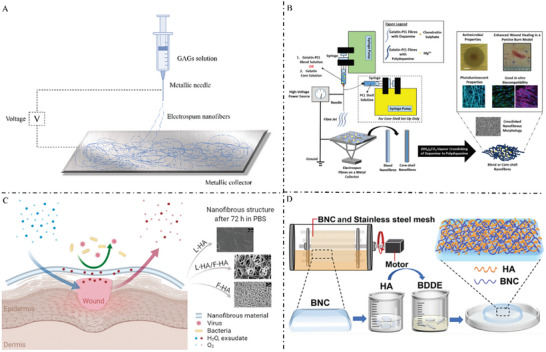
A) Preparation of GAG‐based nanofibers by electrospinning. B) CS and polydopamine cross‐linked electrospun gelatin nanofiber dressings containing mineralized magnesium have antibacterial properties and promote burn wound healing. Reproduced with permission.^[^ ^73^ ^]^ Copyright 2020, Royal Society of Chemistry. C) The nanofiber layer prepared from the hydrophobic derivative of HA can still maintain its fiber structure and dimensional stability after the aqueous medium is relieved, and it can maintain air permeability and good mechanical properties even when dry and wet. Reproduced with permission.^[^ ^105d^ ^]^ Copyright 2021, Elsevier ltd. D) A new bacterial nanocellulose/HA membrane with anisotropic mechanical properties and high light transmission. Reproduced with permission.^[^ ^106^ ^]^ Copyright 2023, American Chemical Society.

GAGs are large linear polymers with abundant reactive groups, which is suitable for forming nanofibers alone or in incorporation with other polymers. Thus, GAG‐based nanofibers have garnered attention in regenerative medicine.^[^ ^105^ ^]^ Magnesium‐mineralized antimicrobial nanofiber dressings containing CS exhibit antibacterial properties and promote burn wound healing by enhancing re‐epithelialization, wound closure, and clinical outcomes.^[^ ^73^ ^]^ Anisotropic scaffolds tailored to different soft tissues of the body can be constructed to guide the alignment of cells during tissue or organ healing by controlling the morphology and alignment of the fibers. For example, A kind of artificial cornea constructed by introducing HA into bacterial nanocelluloses has excellent anisotropic mechanical properties, good cellular compatibility and high moisture retention.^[^ ^106^ ^]^ The wound‐healing effects of such GAG‐based nanofibers have been displayed in **Figure** **7**.

## 4 Role of GAG‐Based Artificial Biomaterials

The essence of wound healing is the creation of new biological skin tissue to replace damaged tissue. As GAGs participate in various biological activities in the tissue microenvironment, cell behavior, and intramolecular interactions, GAG‐based artificial biomaterials are used as carriers and templates for cells and neonatal tissues to provide promising biomaterial approaches for wound healing and even direct construction of artificial skin to improve the repair quality of severe wounds. **Table** **2** lists the common applications and advantages of GAG‐based artificial biomaterials.

**Table 2:** Mechanisms of GAG‐based artificial biomaterials in promoting wound healing (ECM, extracellular matrix)

Clinical applications	Mechanism of action	Key characteristic	Represent of the latest research	References
Barrier/protection of the wound	Against bacterial incursions and infections; Modulates body temperature; Expels bodily fluids.	Superior biocompatibility; Self‐degradability; Barrier protection.	Snail glycosaminoglycan double network biomaterial hydrogel.	[107]
Regenerative microenvironment regulator	Protecting the wound site from potential obstacles; Orchestrate cellular behavior.	Superior biocompatibility; Significant pro‐healing effect; Natural components of the ECM.	Chitosan/glycosaminoglycan scaffolds.	[108]
Bioactive carrier	Enhance or obstruct the communication of an encumbered protein; The intrinsic components that exist within biological tissues.	Superior biocompatibility; Rich presentation forms; Significant pro‐healing effect.	Collagen/hyaluronan based hydrogels.	[109]

### 4.1 Wound Barrier Protector

The skin, which is the largest organ in the human body, serves as an effective shield against bacterial invasion and infection, regulates body temperature, and discharges bodily fluids.^[^ ^110^ ^]^ Medical wound dressings serve as a temporary shield for the wound and play a crucial role in preventing microbial infection, preserving the normal function of tissue cells, facilitating the process of wound repair and tissue regeneration, and promoting clinical wound repair.

**Figure 8:**
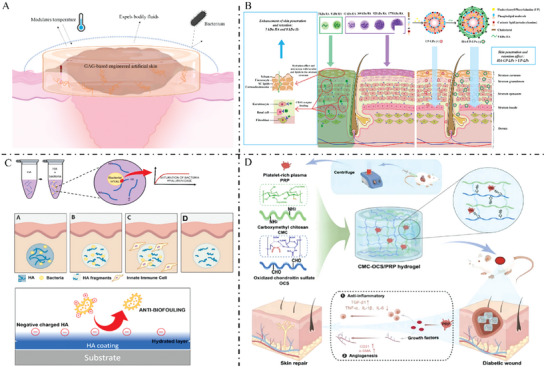
A) GAG‐based biomaterials can simulate the protective effect of skin on the body. B) LMWHA can cross the cuticle barrier into the epidermis and dermis and interact with keratin and lipids in the cuticle barrier to play a role in skin hydration. Reproduced with permission.^[^ ^112^ ^]^ Copyright 2023,Elsevier ltd. C) HA can reduce the bacterial proliferation rate by saturating the bacterial hyaluronidase, prevent the bacterial from attaching to the substrate (anti‐adhesion/anti‐adhesion substrate), or inhibit the bacterial proliferation by repelling the positively charged cell wall of the bacteria. Reproduced with permission.^[^ ^113^ ^]^ Copyright 2023, KeAi Communications Co. D) The nonspecific tissue adhesion hydrogel formed by oxidized CS and carboxymethyl chitosan with dynamic cross‐linking structure can be injected to protect irregular wound surfaces and promote granulation tissue formation, collagen deposition, and angiogenesis. Reproduced with permission.^[^ ^115^ ^]^ Copyright 2023, Elsevier ltd.

GAGs are frequently used to create various artificial skin types, providing protection against further infections or damage. Most GAG‐based biomaterials are hydrophilic polymers that form stable cross‐linked structural networks after absorbing large amounts of water, thereby providing physical isolation and moisture retention properties.^[^ ^111^ ^]^ For example, HA is commonly used in topical preparations because of its superior moisturizing properties and potential to enhance the skin permeability of drugs. LMWHA can penetrate the cuticle barrier into the epidermis and dermis and interact with keratin and lipids in the stratum corneum to provide substantial skin hydration.^[^ ^112^ ^]^ Additionally, the plasticity of GAG‐based biomaterials provides tunable physical and mechanical properties to achieve their structural integrity and prevent microbial invasion.^[^ ^113^ ^]^ For example, A moldable HA hydrogel enabled by dynamic metal‐bisphosphonate coordination that can be used to fill irregularly shaped wound defects without pre‐shaping, Its “ready‐to‐use” plasticity is used to completely protect the wound from further contamination.^[^ ^114^ ^]^ **Figure** **8** summarizes the barrier‐protective effects of GAG‐based biomaterials on the wound surface.

### 4.2 Regenerative Microenvironment Regulator

**Figure 9:**
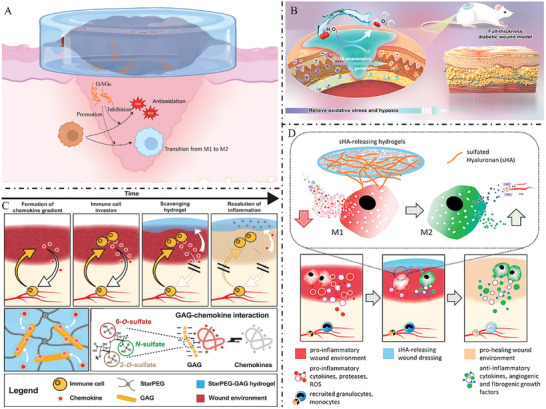
A) GAG‐based materials promote the polarization of macrophages to M2 and antioxidant response to promote wound healing. B) Catalase‐mimic nanozyme‐enhanced hydrogels derived from natural polymers (hydrazide‐aldehyde‐modified HA) and metal‐organic frameworks can capture endogenous elevated ROS in diabetic wounds. Reduce excessive inflammation, effectively induce proliferation, re‐epithelialization, collagen deposition, and neovascularization, to promote diabetic wound healing. Reproduced with permission.^[^ ^121^ ^]^ Copyright 2022, Wiley‐VCH. C) The modular hydrogel of star‐polyethylene glycol and HEP derivative reduces the migratory activity of human monocytes and polymorphonuclear neutrophils by removing inflammatory chemokines from patients with chronic ulcers and alleviates the impairment of inflammatory chemokines on skin wound healing. Reproduced with permission.^[^ ^120a^ ^]^ Copyright 2017, American Association for the Advancement of Science. D) Collagen/hyaluronan based hydrogels are used to modulate the effect of inflammatory macrophage activity on wound healing by releasing sulfated HA as an immunomodulatory component. Reproduced with permission.^[^ ^120b^ ^]^ Copyright 2021, KeAi Communications Co. GAG, glycosaminoglycans; ROS, reactive oxygen species

In addition to protecting the wound bed from potential risks of infection and water loss, GAG‐based biomaterials also manipulating regeneration microenvironment to controlling cellular behaviors.^[^ ^8^ ^]^ Wound healing is orchestrated by the cellular microenvironment. However, due to the damage and destruction of skin structure and function, the wound microenvironment is seriously unbalanced, and deep and large surface area injuries such as deep burns are almost not allowed to heal on their own.^[^ ^116^ ^]^ Inspired by the special physicochemical properties and biological activities of GAGs, including GAGs in biomaterials to construct biomimetic materials is a promising approach for improving regenerative processes via creating a specific regeneration microenvironment.^[^ ^117^ ^]^ Such as the high hydrophilic GAGs give materials based on GAGs the ability to absorb exudates, soften necrotic tissue during wound healing, and create a favorable wound‐healing environment.^[^ ^118^ ^]^ In aqueous solutions, polyanionic GAG attracts divalent cations, such as Ca^2+^ and Na^2+^, resulting in a high hydrodynamic volume and low compressibility, forming a size‐selection barrier. Consequently, only small molecules can diffuse freely, which limits the bioavailability of larger molecules.^[^ ^119^ ^]^ Also, utilizing the ability of HEP to capturing chemokines, researchers customized a modular hydrogel that effectively removed inflammatory chemokines, including monocyte chemokine protein 1(triggering IL‐8), macrophage inflammatory protein 1α, and macrophage inflammatory protein 1β, to reduce inflammation and promote granulation tissue formation, vascularization, and wound healing.^[^ ^120^ ^]^ It is worth mentioning that the biomaterials based on non‐human endogenous GAGs components cannot be ignored, of which excellent biological efficacy in human wound healing has great contributions and prospects. For example, a biomaterial hydrogel with a double‐mesh structure composed of snail GAG and methacrylate gelatin exhibits superior adhesion and biocompatibility. This allows it to effectively mimic the skin, providing a protective covering over wounds, considerably reducing inflammation by isolating pro‐inflammatory cytokines, and promoting wound healing through the inhibition of NF‐ĸB signaling pathway expression, thereby encouraging macrophage polarization towards the M2 phenotype.^[^ ^107^ ^]^ In summary, the function of GAGs in regulating the wound‐healing microenvironment renders GAG‐based biomaterials more comprehensive and safer than conventional dressings.^[^ ^120b^ ^]^ **Figure** **9** illustrated several GAG‐based biomaterials that regulate the wound microenvironment and promote wound healing.

### 4.3 Bioactive Carrier

GAG‐based materials offer unique biological advantages over synthetic materials for the loading and delivery of bioactive molecules, including small molecules and hydrophobic compounds. GAG‐based biomaterials can be physically fabricated into different forms with varying mechanical properties to suit different needs or can be used as a component in a bulk scaffold that provides ideal mechanical and structural properties. Furthermore, these biological materials can provide supplementary functions.^[^ ^122^ ^]^ Thus, a range of biomaterials based on GAGs including hydrogels, aerogels, microneedles, and nanoparticles (Section 3) have been developed for spatiotemporal delivery of active molecules. First, GAGs can either enhance or inhibit the signaling of a loaded protein by acting as cofactors or through sequestration, respectively. HS can act as a receptor to regulate physiological signals by binding to PDGF, FGF, and VEGF, and others, and by acting on fibroblasts, keratinocytes, and epithelial cells to control physiological behaviors, such as angiogenesis, wound contraction, granulation tissue formation, and epithelial regeneration.^[^ ^123^ ^]^ For example, a nanofiber dressing functionalized with HEP utilizes the chelating ability of exogenous and endogenous growth factors to synergistically drive tissue regeneration. The loading efficiency of the prepared nanofiber dressing for an exogenous basic FGF was 80%, and it sequestered 15‐fold more endogenous VEGF. Unlike dressings without HEP functionalization, nanofiber dressing reduces wound inflammation, minimizes scarring in newly formed granulations, and substantially increases the number of newly formed skin cells.^[^ ^124^ ^]^ second, GAG‐based materials not only have the potential to release bioactive molecules, but as carriers themselves can also accelerate the wound healing process.^[^ ^109^, ^125^ ^]^

**Figure 10:**
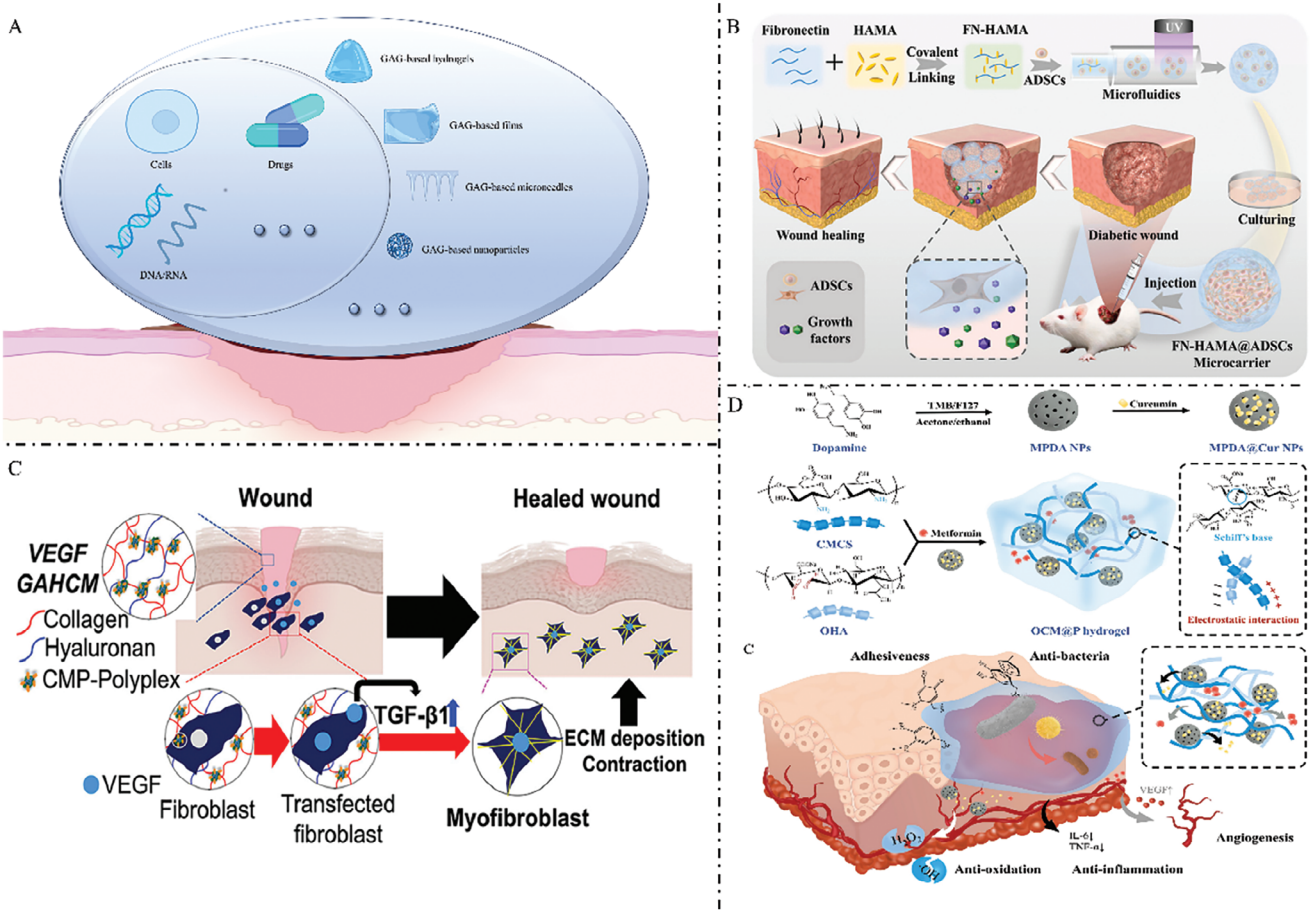
A) GAG‐based materials can be loaded with various active molecules to promote wound healing. B) Hydrogels loaded with adipose‐derived stem cells synthesized by conjugation of HA methacryloyl group to fibronectin molecular chain have great potential for the treatment of diabetic wounds. Reproduced with permission.^[^ ^127^ ^]^ Copyright 2023, KeAi Communications Co. C) HA‐collagen based hydrogel materials loaded with growth factors enhance wound‐healing effects by combining controlled and local growth factor expression and matrix‐mediated regulation of cell behavior. Reproduced with permission.^[^ ^125a^ ^]^ Copyright 2022, Elsevier ltd. D) Hydrogel dressings fabricated from a polymer matrix formed by dynamic imine bonds and electrostatic interactions between carboxymethyl chitosan and oxidized HA, curcumin‐loaded mesoporous polydopamine nanoparticles and metformin, whose multifunctional synergy greatly promotes diabetic wound healing. Reproduced with permission.^[^ ^125b^ ^]^ Copyright 2023, Elsevier ltd.

In addition to deliver active molecules, GAG‐based materials are friendly to a variety of living cells, and serving as excellent carriers for cell transplantation. For example, hydrogels with HA methacryloyl conjugated to the fibronectin molecular chain can be loaded with adipose‐derived stem cells to promote wound healing in diabetes. DS can be used in combination with CS to support chondrocytes that differentiate mesenchymal stem cells.^[^ ^126^ ^]^ These hydrogels have shown considerable effects in promoting new blood vessel formation, hair follicle regeneration, and collagen deposition.^[^ ^127^ ^]^ **Figure** **10** illustrated the functions of GAG‐based biomaterials as bioactive carriers.

## 5 Conclusions and Perspectives

In summary, natural GAGs and their derivatives have been widely utilized in tissue engineering and regenerative medicine, and encouraging results have been achieved in recent years. Nevertheless, biological systems have optimized their organizational hierarchies over billions of years of evolution and natural selection to adapt to complex environmental changes. Consequently, they possess unique properties that are typically difficult to achieve with synthetic materials. Moreover, artificially synthesized biomaterials are not entirely compatible with natural biomaterials in terms of physical and chemical properties or biological effects. Thus, challenges remain for commercialization of GAG‐based biomaterials.

The diverse sources of natural GAGs contain many new potential candidates for wound dressings, but more studies are needed to further explore their structure and biological properties in the future to develop medically promising biomaterials. Currently, most of the sources of GAG‐based biomaterials studied are still limited to common categories of GAGs, such as HA and HEP. Thus, more natural GAGs with good biocompatibility and proregenerative properties require further exploration. Inspired by the survival characteristics of animals, such as snails and giant salamanders, in the natural environment, researchers have developed natural biological adhesions that can promote wound healing, with GAGs being identified as one of the main active ingredients.^[^ ^128^ ^]^ The performance of natural GAGs in tissue regeneration and repair provides additional possibilities for constructing functional biomaterials. Nevertheless, it is still too early to translate research into biomaterials based on these natural GAGs for clinical applications because their structures and mechanisms have not been fully revealed. Therefore, further basic research on the sources, processing methods, functions, and structures of the new natural GAGs are necessary to construct biomaterials with clinical conversion prospects.

Additionally, precise control of the appropriate material composition, 3D structure, and mechanical strength, to meet the healthy physiological structure of skin and the pathophysiological process of wound healing, are still challenging. Wound healing is a highly dynamic process, therefore, the regulation and guidance of the tissue microenvironment, especially the mechanical microenvironment, on tissue regeneration at different stages has gradually attracted more attentions from researchers. Correspondingly, the mechanical properties of biomaterials, such as stiffness and viscoelasticity, play crucial roles in promoting tissue healing should be taken seriously. For instance, owing to the mechanical sensitivity of T cells, low‐hardness hydrogel dressings are more effective in inhibiting inflammation, whereas high‐hardness hydrogel dressings are more conducive to the production of pro‐inflammatory and anti‐inflammatory cytokines.^[^ ^129^ ^]^ Regarding cell proliferation and cytokine secretion, dressings at 100 kPa were more active in cell migration, gene expression, cytokine secretion, metabolism, and cell cycle progression than dressings at ≈0.5 kPa.^[^ ^130^ ^]^ Moreover, gels with moduli below kilopascals are more conducive to cell implantation and angiogenesis.^[^ ^131^ ^]^ However, biomaterials that regulate mechanical cues are mostly synthetic polymers, and the mechanical properties of GAG‐based biomaterials are largely ignored. Therefore, the construction of GAG‐based intelligent biomaterials based on the unique physical and chemical properties of GAGs in terms of controlling mechanical properties, adapting to the tissue microenvironment, and improving responsiveness to diverse external stimuli is an important research topic in this field.

In conclusion, biomaterials incorporating GAGs will stimulate the specific response of the body by giving the material specific structure and biological functions, to control the self‐improvement and rehabilitation functions of the body, and accomplish the regeneration and reconstruction of human tissues. Although most of these GAG‐based biomaterials discussed above are still far from clinical application, we still believe that GAG‐based biomaterials may lead to new therapeutic interventions from the laboratory to the clinically available user‐friendly products with the advancement of interdisciplinary research of regenerative medicine, glycobiology, and materials science, as well as the breakthroughs of the aforementioned challenges. This review outlines the innate roles and bioactive mechanisms of native GAGs during situ wound healing, as well as the presentation of common GAG‐based biomaterials and the adaptability of application scenarios, which provides comprehensive insights for researchers in the design of translational studies between materials and clinical applications.

## Conflict of Interest

The authors declare no conflict of interest.
